# Commentary: Mendelian randomization analysis identifies circulating vitamin D as a causal risk factor for ovarian cancer

**DOI:** 10.1093/ije/dyw265

**Published:** 2016-11-07

**Authors:** Caroline J Bull, James Yarmolinsky, Kaitlin H Wade

**Affiliations:** ^1^School of Social and Community Medicine, University of Bristol, Bristol, UK; ^2^MRC/University of Bristol Integrative Epidemiology Unit, University of Bristol, Bristol, UK; ^3^IGFs and Metabolic Endocrinology Group, School of Clinical Sciences North Bristol, University of Bristol, Bristol, UK

In this issue of the *International Journal of Epidemiology*, Ong *et al.* present evidence for a causal role of vitamin D in ovarian cancer in 10 065 cases and 21 654 controls within the Ovarian Cancer Association Consortium (OCAC).^1^ Specifically, exposure to lower circulating vitamin D (25-hydroxyvitamin D) through natural genetic variation was positively associated with epithelial ovarian cancer and most strongly associated with high-grade serous ovarian cancer.

**Figure 1.**
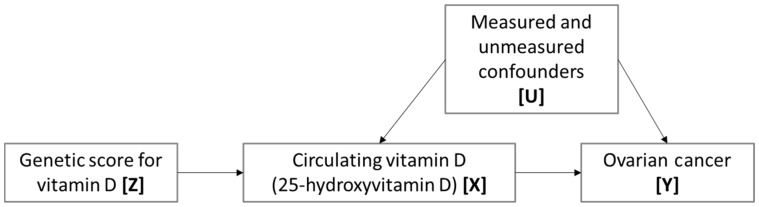
Mendelian randomization: using genetic variants to establish whether circulating vitamin D is causally related to ovarian cancer. An instrumental variable (genetic variation) [Z] acts as a proxy for environmental exposure [X], postulated to influence cancer [Y]. Z is independent of measured or unmeasured confounders [U]. Z only influences Y if the association between X and Y is causal. In a two-sample approach, the association between Z and X is estimated in one sample and the association between Z and Y is estimated in an independent sample.

Few modifiable risk factors have been prospectively associated with ovarian cancer and little is known about its aetiology. Survival rates are poor (< 50% 5 years post diagnosis)^2^, as most patients present with advanced disease which is largely incurable. Vitamin D has received a considerable amount of interest within the field of cancer epidemiology, with a number of observational studies investigating the putative link between circulating vitamin D and ovarian cancer. However, evidence thus far is insufficient to motivate recommendations for vitamin D supplementation.^3^ Further, observational studies are prone to various biases (such as confounding and reverse causation) that can distort observed associations and, although randomized control trials (RCTs) are widely accepted as the gold standard for establishing the effectiveness of an intervention, they are expensive, time consuming and largely unfeasible in a primary prevention setting. Mendelian randomization (MR) is an alternative and increasingly accepted approach to improve causal inference in observational studies.^4^ MR is seeing widespread application in the field of epidemiology and, with regard to vitamin D in particular, recent studies have identified a number of causal associations with health outcomes, including inverse associations with multiple sclerosis^5^ and overall mortality.^6^ Ong and colleagues used a two-sample MR approach to investigate a putative causal association between vitamin D and ovarian cancer (Figure 1).

**Figure 2.**
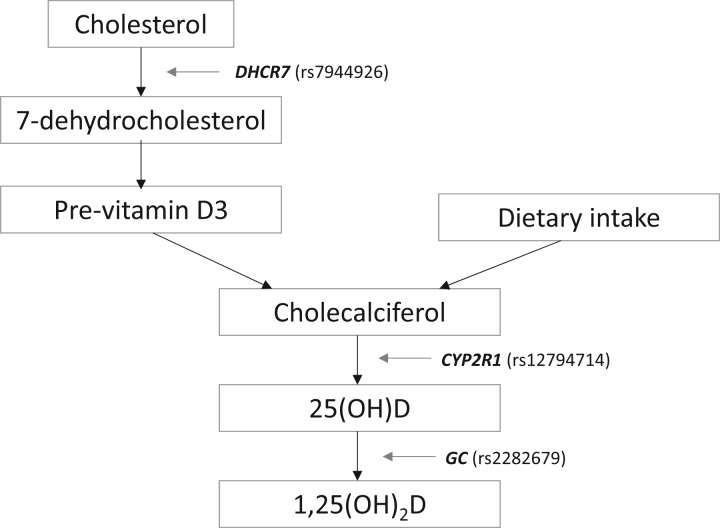
Genetic variation associated with circulating vitamin D levels: rs7944926 is in *DHCR7*, the protein product that converts 7-dehydrocholesterol to cholesterol, reducing its availability for conversion to pre-vitamin D3. rs12794714 is in *CYP2R1*, which is involved in the conversion of cholecalciferol to 25-hydroxyvitamin D [25(OH)D, the measured metabolite] in the liver. rs2282679 is in *GC*, the protein product of which is involved in vitamin D transport and the final conversion of 25(OH)D to the active metabolite 1,25-dihydroxyvitamin D [1,25(OH)_2_].

There are important assumptions that must be considered when using MR to make causal inferences,^4^ which the authors have made efforts to address. First, the genetic score used to instrument circulating vitamin D levels was generated using three single nucleotide polymorphisms (SNPs) from well-characterized pathways involved in vitamin D metabolism (Figure 2.). Although the SNPs together explained only a small proportion of the variance in vitamin D (1.3%), the statistical power and precision of estimates was enhanced by using a large sample size for overall and high-grade serous ovarian cancer (conclusions concerning other subtypes are likely limited, due to relatively few cases). Estimates for the effect of vitamin D on ovarian cancer were scaled to 20 nmol/l in order to make appropriate comparisons with observational estimates, RCTs and previous MR studies.^6–8^ A 20 nmol/l reduction in circulating vitamin D from the 75th centile represents insufficiency^6^ and is therefore clinically interpretable.

Potential associations between individual vitamin D SNPs and possible confounding variables (such as education, smoking status and obesity) were investigated to assess the second assumption of MR. However, regarding potential pleiotropic associations of the SNPs, the authors present an association for two of the three SNPs with height in supplementary analyses. As previous observational analyses have reported a positive association between height and ovarian cancer risk,^9^ this association warrants further discussion. Vitamin D is known to mediate metabolic pathways that influence growth;^10^ therefore, the pleiotropy reported by Ong *et al.* is likely vertical (where the SNP-height association is on the same causal pathway for vitamin D and ovarian cancer). However, horizontal pleiotropy (where the SNP-height association lies on an alternative causal pathway) cannot be confidently ruled out, which may invalidate MR assumptions, drive spurious associations and lead to difficulty in the interpretation of causal estimates. Alternatively, given some evidence to suggest that certain vitamin D pathways may have been selected for by population movement to northern latitudes^11^ and the well-established evidence for selection on height among Europeans,^12^ it is also possible that confounding by population stratification may have contributed to a spurious association between vitamin D SNPs and height. Further work to both confirm an association between circulating vitamin D and height and to investigate evidence for stratification will help to clarify the causal nature of this association.

The authors also investigated a potential pleiotropic association between the vitamin D SNPs and diabetes mellitus. Vitamin D deficiency has been linked previously to diabetes, which itself is associated with ovarian cancer.^13^ However, the authors found no evidence of an association between the SNPs tested and diabetes or measures of glycaemia, using publicly available genome-wide association studies (GWAS) data. In agreement with this, a recent MR study found no evidence to support a causal association between vitamin D and incident type 2 diabetes.^14^

As is done by Ong *et al.*, there is certainly value and transparency in using a few, carefully selected SNPs of well-known biological function as an instrument within an MR analysis. However, given that vitamin D is a highly heritable trait (approximately 53% of variance is explained by genetic variation^15^), further work using additional genetic variants as instruments for vitamin D may increase power in future studies; and applying more recently developed MR methods, such as MR-Egger^16^ and the weighted median approach,^17^ which are sensitivity analyses that enable the detection of horizontal pleiotropy, will help to scrutinize the validity of MR assumptions. Making use of multiple genetic scores that instrument particular components of the vitamin D metabolic pathway,^7^ or screening the genetic score for vitamin D against a number of phenotypic outcomes in a phenome-wide association study (PheWAS),^18^ could help further inform understanding of the aetiology of ovarian cancer and help guide future research.

In summary, Ong *et al.* present evidence for a causal role of low levels of circulating vitamin D in overall and high-grade serous ovarian cancer, using two-sample MR methodology.^1^ Circulating vitamin D levels are modifiable and supplementation may hold potential for ovarian cancer prevention strategies; therefore, further work is needed both to replicate findings presented in this analysis and to help elucidate the mechanisms by which circulating vitamin D may influence ovarian cancer.
